# P05.70. Polypharmacy, interactions and collaborative care in the middle-aged and beyond

**DOI:** 10.1186/1472-6882-12-S1-P430

**Published:** 2012-06-12

**Authors:** M Stargrove, L Stargrove

**Affiliations:** 1 Oregon College of Oriental Medicine, Portland USA

## Purpose

The popular perception of herb-drug and drug-nutrient interactions is that they are unintended and problematic. Actually, purposeful coadministration of therapeutic agents within a clinical strategy represents a valuable approach to complex and evolving patient-centered care. As individuals age, they are more likely to be prescribed pharmaceutical medications: many also take herbs and nutrients, which may interact. The occurrence of polypharmacy may be orchestrated or unsupervised, beneficial or risky, depending upon the individual patient’s physiology, health status and medical condition(s) and the communication and collaboration among the involved healthcare providers.

## Methods

A literature review was conducted using the following keywords: polypharmacy, herb-drug interactions, nutrient-drug interactions, safety, herbs, vitamins, minerals, amino acids, essential fatty acids, physiologics, nutraceuticals, nutritional supplements, adverse drug events, drug-induced nutrient depletion, integrative healthcare delivery, trans-disciplinary models, collaborative care, multidisciplinary care, therapeutic strategies, patient self-care, patient-centered medicine, aging, middle-aged, elderly.

## Results

The challenges of multidisciplinary care involving diverse philosophical and clinical traditions bring forth the opportunity and necessity of formulating new models of proactive polypharmacy focused on patient safety, therapeutic efficacy and evidence-informed clinical-decision making. Patient demographics of medication, herb and nutrient usage, adverse drug event occurrence, and typical risk patterns are reviewed. Cardiovascular, degenerative bone and mind/mood conditions illustrate specific areas of concern.

## Conclusion

Within the standard literature of pharmacy practice, several clinical management tools have been developed for eliciting, assessing, and managing multiple prescribed and over the counter drugs. By extending the tools for coordinating polypharmacy to encompass the full range of therapeutic agents, whether pharmaceutical, nutrient or botanical, clinicians can formulate and implement a patient-centered model of safe and effective polypharmacy.

